# Closed Genome Sequences of Providencia alcalifaciens Isolates from Dogs

**DOI:** 10.1128/mra.00955-21

**Published:** 2022-02-17

**Authors:** Thomas H. A. Haverkamp, Håkon Kaspersen, Øivind Øines, Bjørn Spilsberg, Amar Anandrao Telke, Karin Lagesen, Jannice Schau Slettemeås, Solveig Sølverød Mo, Hannah Joan Jørgensen, Camilla Sekse

**Affiliations:** a Department of Analysis and Diagnostics, Norwegian Veterinary Institute, Ås, Norway; b Department of Animal Health and Food Safety, Norwegian Veterinary Institute, Ås, Norway; Loyola University Chicago

## Abstract

Eight Providencia alcalifaciens isolates from eight different dogs in Norway with acute hemorrhagic diarrhea were sequenced. Based on Illumina and Oxford Nanopore Technologies sequencing, all of the genomes were complete and closed after hybrid assembly.

## ANNOUNCEMENT

In the autumn of 2019, there was an outbreak of acute hemorrhagic diarrhea syndrome in dogs in Norway. Providencia alcalifaciens was identified as a possible cause of the outbreak (1). To determine whether P. alcalifaciens could stem from a common source, isolates from diseased dogs were sequenced. Phylogenetic analysis revealed that several isolates clustered closely together, thus suggesting a common origin (1). P. alcalifaciens has previously been associated with cases of enteritis in dogs (2, 3), but a possible role of this pathogen in causing acute hemorrhagic diarrhea in dogs is not well documented.

We aimed to characterize P. alcalifaciens genomes from some of the affected dogs. In total, eight P. alcalifaciens isolates from eight different dogs were selected for Illumina and Oxford Nanopore Technologies (ONT) sequencing. Here, we report hybrid assemblies of these isolates and characterization of the assemblies.

P. alcalifaciens was isolated from stool or gastrointestinal samples from dogs, as described by Jørgensen et al. (1). Samples were obtained with owner consent, which includes a general clause providing permission to use the samples and the secondary material for research. DNA was extracted on Genomic-tip 100/G columns (Qiagen) using the supplier’s bacterial protocol. The DNA concentration was determined using the Qubit double-stranded DNA (dsDNA) BR assay kit (Thermo Fisher Scientific), and DNA quality was assessed using the NanoDrop One spectrophotometer (Thermo Fisher Scientific). Libraries for Illumina sequencing were prepared using the Nextera Flex library preparation kit (Illumina), followed by sequencing on a MiSeq system (Illumina) at the Norwegian Sequencing Center, using 300-bp paired-end chemistry. High-quality DNA (∼400 ng) from each sample was used for ONT library preparation using the rapid barcoding library preparation kit (SQK-RBK004; ONT) and was indexed using RB1-12 barcodes. Pooled libraries were cleaned with an AMPure XP bead cleanup step. The barcoded library (10 μL) was loaded onto a FLO-MIN106 R9 flow cell on a MinION device (ONT) and run for 37.5 h. Raw sequence data were base called separately after the run using Guppy v.3.4.5 (ONT) and demultiplexed using qcat v.1.1.0 (ONT) (https://github.com/nanoporetech/qcat). The sequence quality of the demultiplexed data sets was checked with NanoPlot v.1.30.0 (4). Default parameters were used for all software unless otherwise specified.

Filtlong v.0.2.0 (https://github.com/rrwick/Filtlong) was used to filter the long reads based on quality. Hybrid assemblies were generated using Unicycler v.0.4.8 (5), followed by Prokka v.1.14.5 (6) to annotate the hybrid assemblies. The GC content of each assembly was calculated using the EMBOSS v.6.6.0 (7) commands union and infoseq. The mean sequencing depth was determined by mapping reads with BWA v.0.7.17 (8) and then calculating the mean coverage using SAMtools v.1.9 (9) depth. MOB-suite v.3.0.1 (10) was used to predict plasmid sequences from the hybrid assemblies and to identify their respective replicon types. Each plasmid FASTA file generated by MOB-suite was examined for the presence of resistance genes, virulence genes, and plasmids by using ResFinder v.4.0 (11), VirulenceFinder v.2.0 (12), and PlasmidFinder v.2.1 (13), respectively. Each isolate was also screened for virulence genes using the web tool VFanalyzer (14), with Escherichia coli as the closest reference organism.

All isolates were complete (100% for all isolates, according to CheckM [15]) and closed after hybrid assembly. No antimicrobial resistance genes were identified. Cytolethal distending toxin B (*cdtB*) was identified on a plasmid in six of the isolates. The characteristics of the genomes, including accession numbers, are presented in Table 1.

**TABLE 1:** Overview of Providencia alcalifaciens genomes

Sample	Part of outbreak cluster	Virulence gene	Size of plasmid related to *cdtB* (bp)	No. of Illumina reads (R1 and R2)	No. of ONT reads	ONT read *N*_50_ (bp)	No. of replicons	Total size (Mbp)	GC content (%)	No. of genes (Prokka)	Coverage (×)	Illumina read accession no.	ONT read accession no.	Assembly accession no.
2019-01-3283-1-1	No	*cdtB*	168,972	679,898	61,938	7,965	2	4,123,814	41.8	3,864	93.0	ERR6686186	ERR6716414	GCA_915401745
2019-04-28369-1-2	Yes	*cdtB*	170,607	751,378	51,255	11,678	5	4,386,911	41.9	4,227	96.2	ERR6688396	ERR6716416	GCA_915401735
2019-04-28370-1-5	Yes	*cdtB*	171,426	723,910	39,773	6,972	6	4,386,930	41.9	4,227	93.7	ERR6688397	ERR6716417	GCA_915401765
2019-04-29290-1-7	No			727,231	69,468	5,291	2	4,218,374	41.8	4,005	97.5	ERR6688399	ERR6716419	GCA_915402005
2019-04-29291-1-1	No			337,335	138,506	4,984	2	3,933,480	42.6	3,607	48.2	ERR6688400	ERR6716420	GCA_915403165
2019-04-29292-1-3	Yes	*cdtB*	171,398	852,192	87,265	8,597	5	4,386,609	41.8	4,229	108.8	ERR6688407	ERR6716421	GCA_915402015
2019-04-27799-1-2	Yes	*cdtB*	171,440	830,802	54,233	13,459	5	4,386,154	41.9	4,229	107.4	ERR6688302	ERR6716415	GCA_915401705
2019-04-29034-1-3	Yes	*cdtB*	171,426	624,173	94,011	10,190	5	4,386,908	41.9	4,228	80.8	ERR6688398	ERR6716418	GCA_915401715

### Data availability.

All data sets have been deposited in ENA under accession number PRJEB47525 (Table 1).
